# An Updated Review on the Relationship of Depressive Symptoms in Obstructive Sleep Apnea and Continuous Positive Airway Pressure

**DOI:** 10.7759/cureus.15907

**Published:** 2021-06-24

**Authors:** Zarmeena Aftab, Adarsh Thomas Anthony, Shermeen Rahmat, Prerna Sangle, Safeera Khan

**Affiliations:** 1 Family Medicine, California Institute of Behavioral Neurosciences & Psychology, Fairfield, USA; 2 Internal Medicine, California Institute of Behavioral Neurosciences & Psychology, Fairfield, USA

**Keywords:** obstructive sleep apnea (osa), depression, anxiety, mood symptoms, psychological symptoms, continuous positive airway pressure (cpap)

## Abstract

Obstructive sleep apnea (OSA) is a common sleep disorder occurring across all age groups, gender, and is multifactorial. The episodic decrease in airflow during sleep results in hypoxia and hypercapnia over time, resulting in morning headache, systemic and pulmonary hypertension, and polycythemia. Fragmentation of sleep at night-time cause daytime somnolence, fatigue, memory problems, and mood symptoms such as depression and anxiety. These secondary mood symptoms could be easily missed by healthcare providers as the primary disorder resulting in unnecessary anti-depressants' prescription. This study investigates the effect of continuous airway pressure (CPAP) on depressive symptoms of OSA. We used PubMed, PubMed Central (PMC), and MEDLINE for data collection. We used OSA, depression, anxiety, mood symptoms, psychological symptoms, and CPAP as the keywords, both alone and in combination. The search ended on November 5, 2020, and it was limited to the year 2010 until the day of the search. However, a few of the papers published earlier than 2010 were also included to have better insight into some aspects of the topic. We included articles measuring the impact of CPAP on mood symptoms using any one of the validated scales, such as the Beck Depression Inventory (BDI), the State-Trait Anxiety Inventory (STAI), Hospital Anxiety and Depression Scale (HADS), or Hamilton Depression Scale (HAM-D). Our initial searches yielded 131 articles. Twenty-one of the 131 papers satisfied the review's criteria. Four studies out of 21 revealed no improvement in OSA-related mood symptoms with CPAP therapy, whereas the others reported beneficial effects on mood, daytime sleepiness, cognition, and patient quality of life.

## Introduction and background

Globally, about one billion people between the ages of 30 years and 69 years, either symptomatic or asymptomatic, are estimated to diagnose with obstructive sleep apnea (OSA) based on the apnea/hypopnea index (AHI) [1]. In 2015, the expense of diagnosing and managing OSA in the United States was nearly 12.4 billion dollars [2]. On the other hand, underdeveloped countries have comparatively little understanding of OSA, and diagnosis and treatment are not easily accessible [3]. 

OSA is characterized by recurrent but transient episodes of disrupted breathing during sleep due to the upper airway collapse, which blocks or decreases airflow despite continuous respiratory efforts, leading to hypoxia and periodic wakefulness [4]. Quantitative measurement of OSA can be made by the apnea/hypopnea index (AHI) using polysomnography, which confirms the diagnosis if AHI >5 per hour is present [4]. OSA has multiple risk factors such as obesity, short/wide neck, neuromuscular disorders, enlarged tonsils/tongue, deviated nasal septum and family history, etc. However, it is highly prevalent in the elderly, men, and obese [5]. 

**Figure 1:**
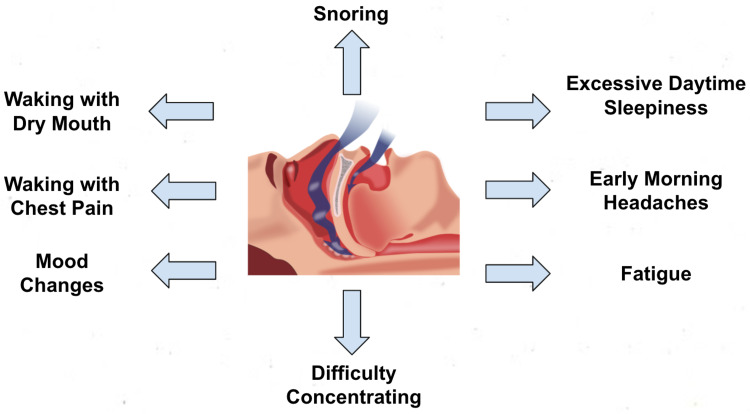
Symptoms of OSA Image created by the author (Zarmeena Aftab). OSA: obstructive sleep apnea

The patients often complain of heavy snoring and constant wakening from sleep, gasping for breath, mainly heard by their sleeping partner. Inadequate sleep at night leads to daytime sleepiness, headache, and impaired concentration/attention, which affects the patient's quality of life and a potential cause of traffic-related accidents as shown in Figure 1. In OSA, depression is quite prominent, with a prevalence of up to 63% reported [6], as both diseases have overlapping symptoms of sleeplessness, exhaustion, irritable mood, and memory impairment [7]. Consequently, patients with depressive OSA symptoms are often diagnosed with depression and treated with anti-depressant before being considered for sleep disorder [8]. 

CPAP is the recommended treatment of choice. It works by constantly forcing air pressure through the patient's mouth and nose throughout the entire respiratory cycle and opening the upper airways mechanically, thus reversing the symptoms of OSA [9]. When used properly at the required pressure, it results in a better sleep cycle and daytime symptoms. It helps lower blood pressure and heart rate by reducing sympathetic activity, resulting in improved cardiovascular risks [10]. Though CPAP is shown to be beneficial in alleviating different adverse effects of OSA, there is a knowledge gap in CPAP's effectiveness on the main risk factor of OSA, i.e., obesity. 

There are several studies conducted so far to see the effect of CPAP on mood symptoms of OSA. Many showed a positive effect on the depressive symptoms of OSA, while others showed little effect. We performed this narrative review by following the scale for the assessment of narrative review articles (SANRA) guidelines. This review aimed to investigate whether CPAP therapy improves depression in patients with OSA so that people with mood symptoms of OSA are treated with CPAP before they are wrongly prescribed anti-depressants. 

## Review

OSA is a prevalent condition with several medical and psychological complications. The purpose of this study was to assess the effects of nocturnal CPAP breathing on the emotional status and cognitive performance in OSA patients. A total of 21 articles were reviewed for this study [11-30]. To find, screen, and choose relevant publications, we used PubMed, PubMed Central (PMC), and MEDLINE as our primary databases. We selected papers that were both relevant to the topic and published in English. Unpublished or gray literature was excluded.

Effect of duration and adherence of CPAP on mood symptoms of moderate-to-severe OSA

Many studies monitored the effect of CPAP on mood symptoms of OSA over a brief period, which was, on average, not more than three months. Although the data are limited regarding the long-term effect and adherence of CPAP on the sustainability of mood in OSA, we were able to find some papers that are discussed below. 

A longitudinal cohort study on 365 patients with moderate to severe OSA conducted by Sin et al. showed an improvement in short-term and long-term well-being and quality of life by using CPAP [11]. While the effect on depressive symptoms was not explored, it was noted that patients demonstrated sustained improvement in emotional summary scores and vitality scores (as obtained by using Medical Outcomes Research Short Form, SF-36) at three months and one-year follow-up. This study also highlighted that patients with the most severe OSA (AHIs ≥ 40) gained the greatest benefit [11]. Similarly, studies by Platon et al. and Schwartz and Karatinos showed a significant improvement in the depression of severe OSA with 11-14 months of therapy with CPAP, however, they both had a small sample size of just five patients and fifty patients, respectively [12,13]. On the other hand, Borak et al. were unable to identify any change in psychological symptoms with one year of nasal CPAP on a sample of twenty patients. Nevertheless, they observed substantial improvement in cognitive functions such as attention and memory [14]. It is worth noticing that these researchers did not monitor the proper use of CPAP by themselves in all the studies mentioned above. It is unclear if it would have affected the outcome of the studies. Furthermore, it is unknown if the use of different scales of depression and anxiety could have affected the results. 

Regarding the effect of CPAP compliance, Lundetrae et al. observed a significant improvement in depression and anxiety in OSA patients using the Hospital Anxiety and Depression Scale (HADS). They followed patients at 20 weeks and noticed that reduction in depression with CPAP use was associated with adherence to its use (>4 hours per night), while no such interaction was found for anxiety score [15]. They further indicated that one of the causes for non-compliance with CPAP therapy might be stress and anxiety, making people less compliant with CPAP [15]. Thus, more trials are needed to determine whether psychiatric disorders can impact compliance with the OSA. The greatest strength of this research was the large sample size of around 468 participants and the use of a well-validated scale for depression and anxiety, which, unlike other scales, did not contain questions of vegetative symptoms of OSA such as exhaustion and sleepiness. Lastly, a randomized controlled trial (RCT) by Engleman et al. showed notable amelioration in attention, daytime somnolence, and emotional symptoms in patients who were adherent with CPAP use [16]. They detected that daytime outcome was correlated with the night-time frequency of microarousals, which depended on the appropriate usage of CPAP during sleep. This analysis provided evidence that compliance with CPAP results in better treatment response. Furthermore, Engleman et al. highlighted a relative inconvenience with CPAP therapy as only 41% of patients preferred CPAP [16]. Table 1 demonstrates the results of studies that are discussed above.

**Table 1:** Effect of Duration and Adherence of CPAP on Mood Symptoms of Moderate-to-Severe OSA CPAP: continuous positive airway pressure, OSA: obstructive sleep apnea, NCPAP: nasal continuous positive airway pressure

Author	Year of publication	The objective of the study	Conclusion/results
Sin et al. [11]	2002	To determine the effect of CPAP on the health status of patients with OSA.	CPAP was effective in improving the health-related quality of life of patients with OSA.
Platon et al. [12]	1992	To evaluate the psychological disturbances in OSA and the impact of NCPAP on these symptoms.	Severe OSA was associated with serious psychological disturbances that improved with NCPAP.
Schwartz and Karatinos [13]	2007	To determine if CPAP provides a sustainable improvement in OSA depressive symptoms.	There was sustained improvement in depressive symptoms who continued to use CPAP.
Borak et al. [14]	1996	To determine the effect of CPAP in patients with serious OSA on mental and cognitive status.	CPAP was effective in improving the cognitive functions of the patients but not emotional status.
Lundetrae et al. [15]	2020	To observe the effect of CPAP on depression and anxiety in patients with OSA.	There was a reduction in anxiety and depression symptoms in those who were adherent with the CPAP use.
Engleman et al. [16]	1999	To evaluate the impact of CPAP on daytime symptoms of OSA patients.	In patients with OSA, the use of CPAP improved daytime symptoms.

Effect of CPAP on daytime function, sleep, and cognition

As a result of night-time sleep fragmentation in OSA, many patients also experience other subjective symptoms associated with depression, such as daytime sleepiness, tiredness, impaired memory, and concentration. 

Engleman et al. evaluated daytime functions by assessing sleepiness, cognitive performance, and overall well-being using various scales. This study discovered that CPAP was effective in improving quality of life, and cognitive functions after four weeks of CPAP but failed to improve subjective and objective sleepiness, which might be attributed to their shorter duration trial [17]. Csábi et al. conducted a clinical trial of 24 recently diagnosed and untreated patients who underwent CPAP therapy for two and a half months. They concluded that CPAP helps restore normal sleeping patterns by improving breathing during sleep, reducing sleepiness's subjective sensation [18]. Furthermore, they also found a major increase in short-and long-term verbal memory and complex working memory which is also supported by Borak et al. when CPAP was used for one year [14,18]. The above-mentioned studies had a small sample size that was overcome by Bhat et al., who observed 182 respondents. They found a rather interesting finding in their research, that while there was an improvement in the subjective symptoms of daytime sleepiness, exhaustion, and depression in both mild and severe form of OSA, objective vigilance was found to be improved only in severe OSA as assessed by the psychomotor vigilance task (PVT) [19]. However, no relationship was found between subjective and objective complaints, and they believed that they are distinct symptoms of the same disorder and should be assessed separately [19]. Table 2 further elaborates the studies done to evaluate the effect of CPAP on various subjective symptoms of OSA.

**Table 2:** Effect of CPAP on Daytime Function, Sleep, and Cognition CPAP: continuous positive airway pressure, OSA: obstructive sleep apnea

Author	Year of publication	The objective of the study	Conclusion/results
Engleman et al. [17]	1997	To evaluate if CPAP improves symptoms and daytime functions in patients with mild OSA.	In patients with OSA, CPAP improved symptoms and daytime functions.
Csábi et al. [18]	2012	To assess the impact on sleep patterns, cognitive function, and anxiety with short-term CPAP use.	Two and a half months of CPAP use improved respiration during sleep, sleep pattern, and subjective sleepiness.
Bhat et al. [19]	2018	To examine the association between subjective improvements in daytime sleepiness, fatigue, and depression in patients with OSA and objective vigilance using CPAP.	In OSA patients, no correlation was identified between reduced subjective symptoms and objective vigilance with the use of CPAP

Effect of CPAP on depression, anxiety, and quality of life in OSA patients 

We ensured to include studies that measured the effect of CPAP on depression and anxiety by using any one of the validated scales such as the Beck Depression Inventory (BDI), the State-Trait Anxiety Inventory (STAI), Hospital Anxiety and Depression Scale (HADS), or Hamilton Depression Scale (HAM-D). Five observational studies found that CPAP improves OSA's psychological complaints when used over three months [20-24]. One study even finds it to be more effective in improving depressive symptoms than surgical treatment [20]. Similarly, before receiving therapy with CPAP, Sanchez et al. observed many of their study participants complaining about getting fired from their work, unable to drive, and experiencing difficulties relating to their sexual life, primarily due to night-time sleep fragmentation leading to daytime tiredness [22]. Using the Epworth Sleepiness Scale, Dostálová et al. concluded that the positive effect of CPAP is mostly attributed to a drop in AHI during sleep, which results in an increase in daytime sleepiness and a decline in depressive symptoms [23]. 

In contrast, Mok et al.'s 12-week CPAP study showed no overall improvement in depression scores as measured by the depression, anxiety, and stress scale (DASS) [25]. A meta-analysis conducted by Gupta et al. reviewed 33 studies that constituted 895 participants, found that CPAP is ideal for reducing respiratory symptoms than psychological symptoms [26]. In the meta-analysis of nine RCTs, Yang et al. concluded that CPAP should be used for a minimum of four hours per night to be effective for OSA psychological symptoms [27]. A meta-analysis by Povitz et al. on 19 RCT also showed inconclusive findings [28]. Although they analyzed RCT, the use of different scales and questionnaires could have biased the results. 

In a placebo-controlled trial published in 2007, Haensel et al. discovered that CPAP improves respiratory disturbance index (RDI) only in the therapeutic group [29]. However, as assessed by the Profile of Mood States (POMS) scale, mood symptoms improved equally in both groups, i.e., therapeutic CPAP and placebo-CPAP. They suggested that CPAP's beneficial impact on OSA's mood symptoms may be attributable to the placebo effect because of the patient's trust and belief in the physician rather than CPAP's functional effect. Similarly, Lee et al. performed a double-blind, parallel, randomized controlled trial on 71 patients, found no change in the Center for Epidemiologic Studies-Depression scale (CES-D), POMS depression, POMS tension, Brief Symptom Inventory (BSI) Depression scale, or BSI anxiety scores in either treatment and placebo group after three weeks of treatment (all P>0.05) [30]. But there was a reduction in respiratory disturbance index (RDI) in the group that received therapeutic CPAP. The main limitation of these two trials was the relatively less severe mood symptoms at the baseline might have resulted in the "floor effect" which gives rise to the finding that CPAP is not superior to placebo regarding its effectivity on mood. Moreover, a two-to-three-week period of a trial might not be enough to explore this. While the risk of confounders for mood symptoms of OSA may have been removed by excluding patients with medical diseases besides OSA and hypertension, it might have resulted in a floor effect for which more studies are required which involve patients with greater sleep issues, psychological complaints, and medical diseases to exclude a floor effect. Table 3 shows the lists of studies documenting the effects of CPAP on mood symptoms in OSA patients.

**Table 3:** Effect of CPAP on Depression, Anxiety, and Quality of Life in OSA Patients CPAP: continuous positive airway pressure, OSA: obstructive sleep apnea

Author	Year of publication	The objective of the study	Conclusion/results
Li et al. [20]	2016	To explore short-term and long-term changes in anxiety and depression in patients undergoing different treatments for OSA.	CPAP improved the quality of sleep, quality of life, depression, and anxiety of OSA patients after six months of treatment, while surgical treatment only improved anxiety over the same period.
Çelik et al. [21]	2016	To assess the effects on depression, anxiety, and perceived levels of stress of persistent CPAP use.	CPAP had positive impacts on psychological parameters such as depression, anxiety, and perceived stress.
Sánchez et al. [22]	2001	To determine the effect of CPAP on depression and anxiety in OSA patients.	Both depression and anxiety decreased after CPAP use.
Dostálová et al. [23]	2019	To determine the neurocognitive and neuropsychiatric effects of CPAP in OSA	CPAP use showed alleviation in daytime sleepiness, as well as signs of depression and anxiety.
Edwards et al. [24]	2015	To assess the effect of CPAP on depression in OSA.	Depressive symptoms in OSA were reduced significantly after CPAP use.
Mok et al. [25]	2020	To assess the effect of CPAP on depression in OSA.	Three months of CPAP did not improve depression scores in OSA patients.
Gupta et al. [26]	2016	To assess whether positive airway pressure (PAP) therapy reduces symptoms of anxiety and depression.	The effect of CPAP in patients with OSA on symptoms of depression and anxiety and quality of life was moderate.
Yang et al. [27]	2020	To quantitatively assess whether CPAP therapy improves mood symptoms in OSAS patients.	Treatment with CPAP improved depression in OSA patients.
Povitz et al. [28]	2014	To assess the effect of CPAP on depression in OSA.	The findings of CPAP's effectiveness in improving depression were inconclusive.
Haensel et al. [29]	2007	To assess the effect of CPAP on mood symptoms of OSA.	No specific beneficial impact of CPAP treatment was observed on mood in OSA patients.
Lee et al. [30]	2012	To assess the effect of CPAP on mood symptoms of OSA compared to placebo.	CPAP treatment did not show a specific therapeutic effect on mood symptoms in patients with OSA.

Limitations

The limitation of This study is that most of the studies we analyzed were observational, had smaller sample sizes, and were conducted for a short time period. Therefore, to find more definitive proof of CPAP's beneficial effects on OSA mood symptoms, more RCTs are needed. 

## Conclusions

We reviewed published literature to determine CPAP's impact on mood symptoms in patients with OSA. The studies analyzed from the past 10 years showed that sleep fragmentation and sleep breathing disturbances are partly responsible for OSA's affective changes. The patients typically suffer from subjective complaints, such as daytime sleepiness, exhaustion, and loss of attention, which correlate with psychological conditions such as anxiety and depression. After evaluating the findings, we found that in well-treated OSA patients, CPAP therapy improves the quality of life (QoL) and lessens depressive symptoms and cognitive functions. Although some of the research did not show any change in OSA psychiatric problems with CPAP, several of these studies showed that patients were not adherent to CPAP largely because of the device-related discomfort. The improvement in psychiatric symptoms thus seems to be dependent on adherence to CPAP. We recommend OSA patients should undergo careful screening for depressive disorders and before initiating other treatment modalities for mood symptoms, CPAP should be attempted first. 
